# Supplementary material for: On the implications of aerosol liquid water and phase separation for organic aerosol mass
Source: Atmos Chem Phys. Author manuscript; Available in PMC 2018 Aug 22. (PMC6104851; doi:10.5194/acp-17-343-2017)
Supplement: Supp [file NIHMS982683-supplement-Supp.zip › acp-2016-719-supplement-version4.pdf]

Supporting information for:

On the implications of aerosol liquid water and phase separation for modeled organic aerosol mass

H. O. T. Pye et al.
pye.havala@epa.gov

Supporting information contains: 12 pages
6 tables
3 figures

OM/OC and O:C Unit Conversions

Separation RH

Separation RH (fraction, 0–1) as a function of molar O:C for ammonium sulfate $(NH_4)_2SO_4$ is (You et al., 2013):

$$SRH = 1/(1 + \exp((O:C - 0.68) / 0.10)) \quad (S1)$$

Neglecting the 5 polymer samples (polypropylene glycol, ethylene glycol containing):

$$OM/OC=1.3013 \times O:C + 1.1663 \quad (S2)$$

The r^2 for OM/OC vs. O:C in equation S2 is 0.99.

Kappa (κ)

Original Lambe et al. (2011) fit:

$$\kappa_{org} = (0.18 \pm 0.04) \times O:C_{old} + 0.03 \quad (S3)$$

Following Canagaratna et al. (2015), the O:C ratios obtained from AMS instruments are increased 27% to account for a low bias in old calibrations (Canagaratna et al., 2015):

$$O:C = 1.27 \times O:C_{old} \quad (S4)$$

Thus:

$$\kappa_{org} = 0.14 \times O:C + 0.03 \quad (S5)$$

The relationship between kappa and O:C (improved-ambient) are recast in terms of OM/OC (improved-ambient) using the AMS relationship (Canagaratna et al., 2015):

$$OM/OC=1.29 \times O:C + 1.17 \quad (S6)$$

As a result, Lambe et al. 2011 becomes

$$\kappa_{org} =0.11 \times OM/OC - 0.10 \quad (S7)$$

For $\kappa_{org} =0.37 \times O:C_{old} - 0.09$ (fit based on Jimenez et al. (2009), Raatikainen et al. (2010), and Duplissy et al. (2011)):

$$\kappa_{org} = 0.23 \times OM/OC - 0.35 \quad (S8)$$

Other

When OM/OC needs to be related to O:C and AMS data or a specific molecular structure is not directly involved, we use Simon and Bhave (2012) equation S1 assuming only H, C, and O atoms:

$$O:C=12/15(OM/OC) - 14/15 \quad (S9)$$

Equivalently,

$$OM/OC = 1.25 \times O:C + 1.17 \quad (S10)$$

Which results in OM/OC that agree with the improved-ambient AMS relationship (Canagaratna et al., 2015) within 2%.

Table S1: New deposition surrogates

Species	Henry's Law Coefficient (M/atm)	Diffusivity (cm^2/s)	Relative reactivity	f0 (meso parameter from Wesely)	Lebas molar volume (cm^3/mol)
IEPOX	IEPOX ($3x10^7$)	0.0579	8	0	110.8
HACET	HACET ($2.93x10^3$)	0.106 (Nguyen et al., 2015)	8	0	72.6

Table S2: Revised deposition species

Species	Old dry deposition surrogate	Old wet deposition surrogate	New dry deposition surrogate	New wet deposition surrogate
NISOPOOH	H2O2	H2O2	NTRM	HYDROXY_NITRATES (1.7×10^4 M/atm)
HPALD	none	none	OP	hydroxy_peroxide (8.3×10^4 M/atm)
ISOPOOH	OP	HYDROXY_PEROXIDE (H=8.3×10^4 M/atm)	IEPOX	no change

Table S3: Revised surrogate properties

Species	Property	Old Value	New Value	Reference
PROPNN	Henry's Law Coefficient	1×10^3 M/atm	1×10^4 M/atm	Nguyen et al. (2015)
H2O2	Henry's Law	8.3×10^4 M/atm	1.1×10^5 M/atm	Sander (1999)
H2O2	Relative reactivity	30	34,000	Nguyen et al. (2015)
HYDROXY_NITRATES, PROPNN, ORG_NTR	f0 (meso parameter)	0.1	0	Nguyen et al. (2015)
OP	f0 (meso parameter)	0.1	0.3	Wolfe and Thornton (2011)

Table S4: Observed and modeled organic aerosol concentrations at SOAS CTR. Values used in Figure 1. WSOC is converted to organic aerosol using an OM/OC of 2.1.

Species	Model or Observation?	Value [$\mu g/m^3$]
POA	Observation	0.47
POA	Model	0.84
Water soluble OA	Observation	1.993×2.1
Aqueous SOA	Model	0.362×2.1
Total OA	Observation	5.44
Total OA	Model	3.58

Table S5: Domain-wide deposition (in percent) of semivolatiles by phase (gas, aerosol) and type (dry, wet) for each volatility class and overall. Values used in Figure 5.

	C*<0.1 μg/m^3	C*=0.1 μg/m^3	C*=1 μg/m^3	C*=10 μg/m^3	C*=100 μg/m^3	Overall: Base Simulation	Overall: Updated Simulation
Dry, Aerosol	3.7	2.8	1.6	0.2	0.02	0.8	0.8
Wet, Aerosol	96.3	63.4	34.5	4.5	0.7	21.6	20.8
Dry, Gas	0	13.5	28.6	43.6	39.1	27.2	32.2
Wet, Gas	0	20.4	35.2	51.7	60.2	50.3	46.2

Table S6: *A posteriori* properties at 298K used in $\gamma \neq 1$ simulation. MTNO3 Henry's law coefficient was increased by 100x compared to *a priori* estimates (Table 2). Activity coefficients at infinite dilution were decreased by 10x compared to Table 2 resulting in a decrease in C_H^* of 10x. Values different from *a priori* estimates are shaded grey.

	C_0^*	H	OM/OC	$\widetilde{M}$	nC	Dg	V_{Lebas}	κ	C_H^*	γ^{∞}
species	μg/m^3	M/atm	g/g	g/mol	-	cm^2/s	cm^3/mol	-	μg/m^3	-
ALK1	0.1472	6.2E+08	1.56	225	12	0.0514	280.5	0.07	83	565
ALK2	51.8775	4.5E+06	1.42	205.1	12	0.0546	275.6	0.06	10446	201
BNZ1	0.302	2.1E+08	2.68	161	5	0.0642	134.1	0.19	175	579
BNZ2	111.11	2.0E+06	2.23	134	5	0.0726	127.5	0.15	15476	139
BNZ3	0.01	NA	3	180	5	0.0596	138.1	0.23	NA	NA
DIM	0.01	NA	2.07	248.2	10	0.0481	248.1	0.13	NA	NA
GLY	0.01	NA	2.13	66.4	3	0.1159	64.9	0.13	NA	NA
IEOS	0.01	NA	3.6	216.2	5	0.0527	147.8	0.3	NA	NA
IETET	0.01	NA	2.27	136.2	5	0.0718	127.1	0.15	NA	NA
IMGA	0.01	NA	2.5	120.1	4	0.0781	105	0.18	NA	NA
IMOS	0.01	NA	4.17	200.2	4	0.0555	130.3	0.36	NA	NA
ISO1	116.01	4.3E+07	2.2	132	5	0.0733	126.3	0.14	695	6
ISO2	0.617	3.7E+09	2.23	133	5	0.0729	123.8	0.15	8	13
ISO3	0.01	NA	2.8	168.2	5	0.0624	135.9	0.21	NA	NA
ISOPNN	8.9	4.5E+08	3.8	226	5	0.0512	151.9	0.32	114	13
MTHYD	0.01	NA	1.54	185	10	0.0585	233.8	0.07	NA	NA
MTNO3	12	1.5E+08	1.9	231	10	0.0505	248.8	0.11	350	29
OLGA	0.01	NA	2.5	206	7	0.0545	168.2	0.18	NA	NA
OLGB	0.01	NA	2.1	248	10	0.0481	234.8	0.13	NA	NA
ORGC	0.01	NA	2	177	7	0.0603	180.3	0.12	NA	NA
PAH1	1.6598	5.1E+07	1.63	195.6	10	0.0564	235.7	0.08	876	528
PAH2	264.6675	7.2E+05	1.49	178.7	10	0.0599	231.5	0.06	56704	214
PAH3	0.01	NA	1.77	212.2	10	0.0534	239.8	0.09	NA	NA
SQT	24.984	6.2E+08	1.52	273	15	0.0451	346.5	0.07	100	4
TOL1	2.326	4.2E+07	2.26	163	6	0.0637	153.7	0.15	888	382
TOL2	21.277	7.3E+06	1.82	175	8	0.0607	194.1	0.1	5477	257
TOL3	0.01	NA	2.7	194	6	0.0567	159	0.2	NA	NA
TRP1	14.792	9.9E+08	1.84	177	8	0.0603	194.9	0.1	41	3
TRP2	133.7297	1.4E+08	1.83	198	9	0.0559	218.8	0.1	329	2
XYL1	1.314	6.2E+07	2.42	174	6	0.061	154.6	0.17	641	488
XYL2	34.483	4.0E+06	1.93	185	8	0.0585	194.6	0.11	10597	307
XYL3	0.01	NA	2.3	218	8	0.0525	194.1	0.15	NA	NA

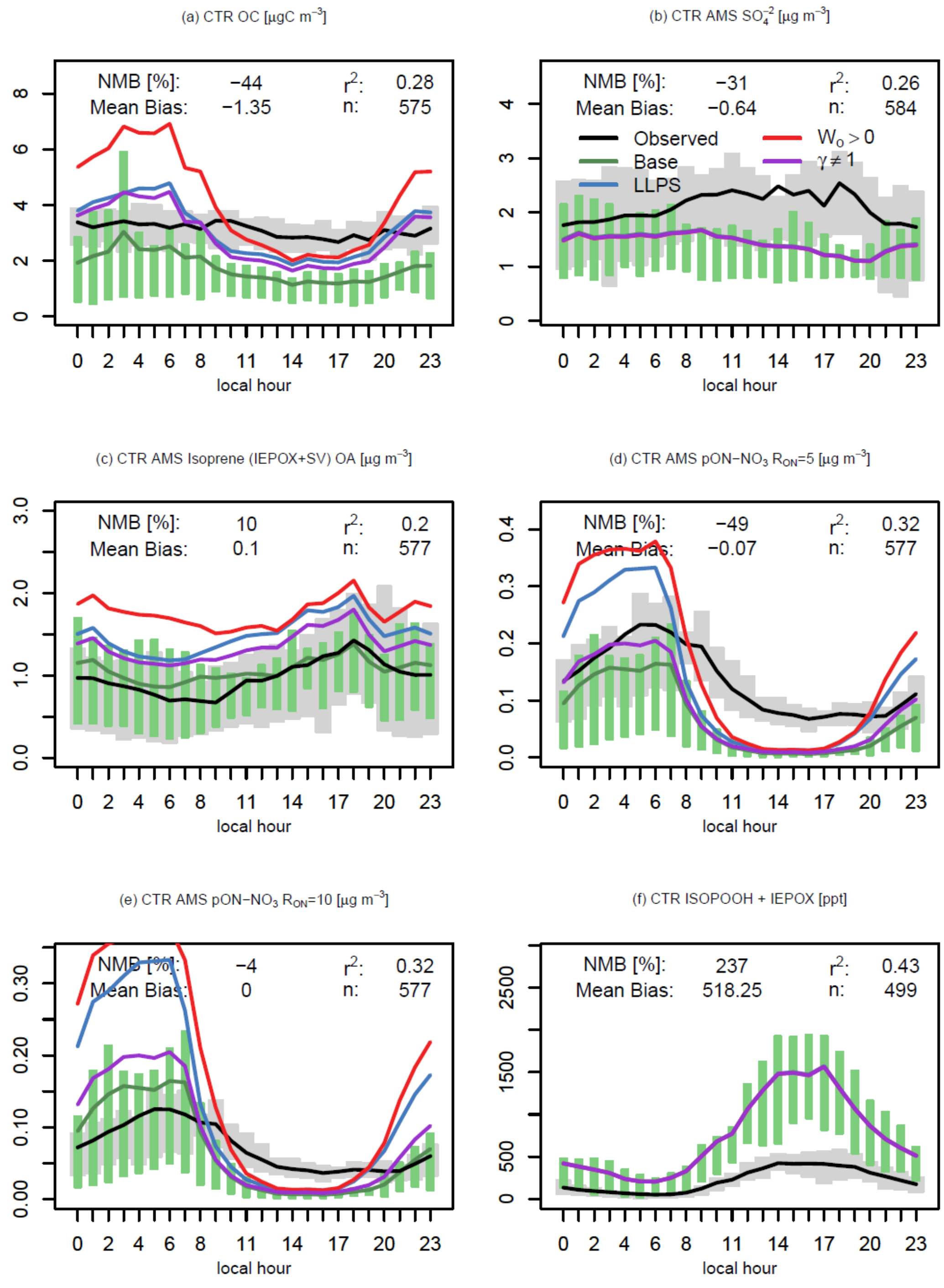

Figure S1: Diurnal variation in modeled and observed species during SOAS. CTR AMS data is from Georgia Tech (Xu et al., 2015). CTR ISOPOOH+IEPOX data is from the CIT-ToF-CIMS (Nguyen et al., 2015). CTR particle-phase tetrol data is from the SV-TAG instrument (Isaacman et al. 2014). Isoprene is from the PTR-MS (Misztal et al. in preparation). OH is from the GTHOS (Feiner et al., 2016). LRK AMS data is from Budisulistiorini et al. (2015). Mean bias, normalized mean bias (NMB), and r^2 refer to the base simulation and observations. Colored lines represent different model simulation predictions. Vertical bars represent interquartile range.

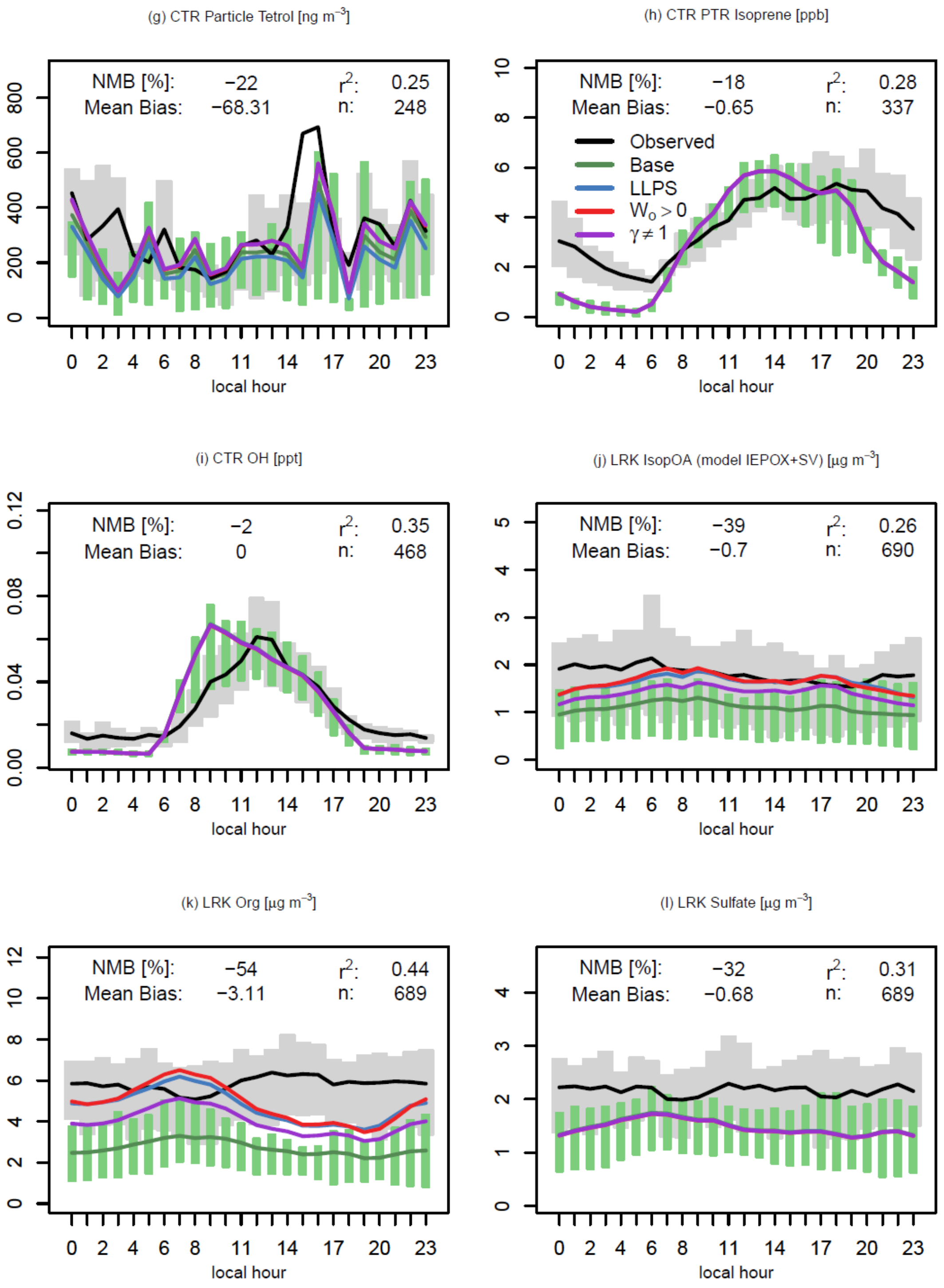

Figure S1 continued

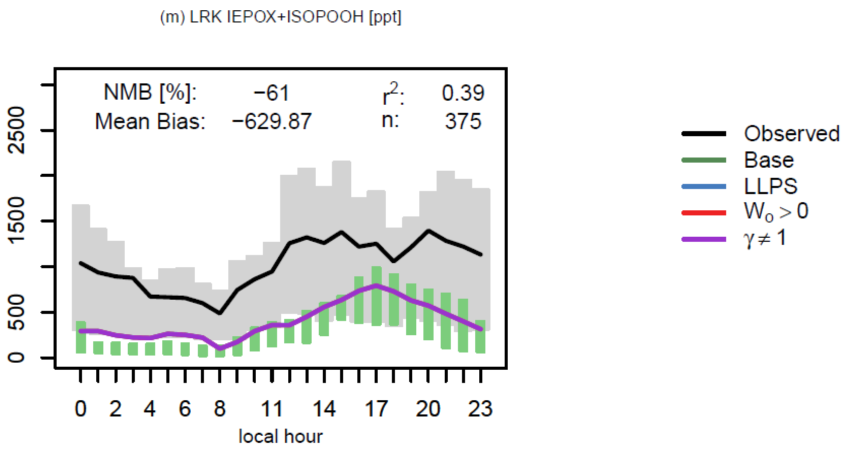

Figure S1 continued

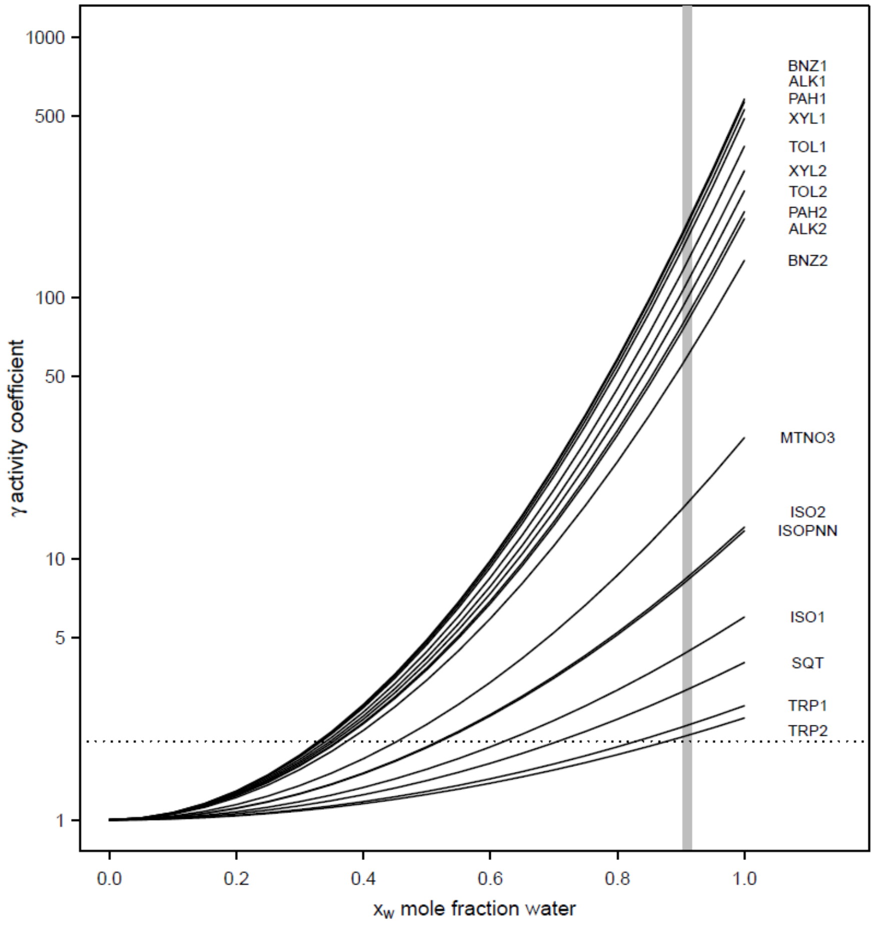

Figure S2: Activity coefficients ($\gamma \neq 1$ simulation) for semivolatile organics as a function of mole fraction water in the particle at 298 K. Dotted line indicates $\gamma = 2$ which would result in a factor of 2 increase in the saturation concentration. The vertical grey line indicates the average mole fraction of water in the particle estimated from SOAS observations (0.91). The MTNO3 Henry's law coefficient has been increased by a factor of 100 compared to *a priori* assumptions. Activity coefficients at infinite dilution ($x_w = 1$) have been reduced by a factor of 10 compared to *a priori* assumptions.

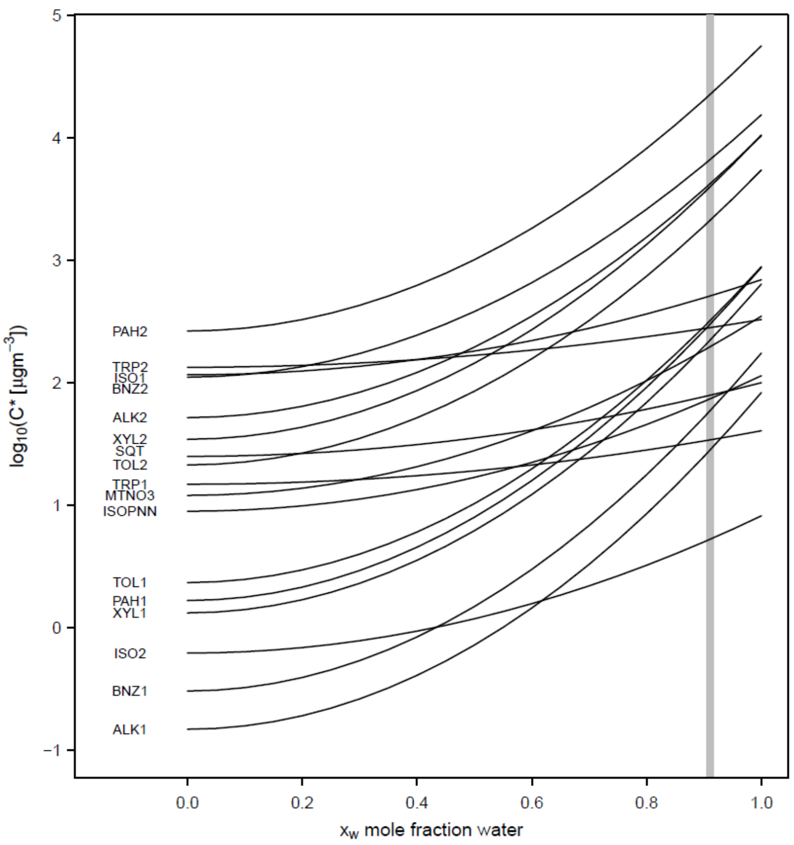

Figure S3: Saturation concentration (C^*_i) as a function of aerosol water mole fraction at 298 K. The vertical grey line indicates the average mole fraction of water in the particle estimated from SOAS observations (0.91). The MTNO3 Henry's law coefficient has been increased by a factor of 100 compared to *a priori* assumptions. Activity coefficients at infinite dilution (x_w = 1) have been reduced by a factor of 10 compared to *a priori* assumptions.

REFERENCES

Budisulistiorini, S. H., Li, X., Bairai, S. T., Renfro, J., Liu, Y., Liu, Y. J., McKinney, K. A., Martin, S. T., McNeill, V. F., Pye, H. O. T., Nenes, A., Neff, M. E., Stone, E. A., Mueller, S., Knote, C., Shaw, S. L., Zhang, Z., Gold, A., and Surratt, J. D.: Examining the effects of anthropogenic emissions on isoprene-derived secondary organic aerosol formation during the 2013 Southern Oxidant and Aerosol Study (SOAS) at the Look Rock, Tennessee ground site, Atmos. Chem. Phys., 15, 8871–8888, doi:10.5194/acp-15-8871-2015, 2015.

Canagaratna, M. R., Jimenez, J. L., Kroll, J. H., Chen, Q., Kessler, S. H., Massoli, P., Hildebrandt Ruiz, L., Fortner, E., Williams, L. R., Wilson, K. R., Surratt, J. D., Donahue, N.M., Jayne, J. T., andWorsnop, D. R.: Elemental ratio measurements of organic compounds using aerosol mass spectrometry: characterization, improved calibration, and implications, Atmos. Chem. Phys., 15, 253–272, doi:10.5194/acp-15-253-2015, 2015.

Duplissy, J., DeCarlo, P. F., Dommen, J., Alfarra, M. R., Metzger, A., Barmpadimos, I., Prevot, A. S. H., Weingartner, E., Tritscher, T., Gysel, M., Aiken, A. C., Jimenez, J. L., Canagaratna, M. R., Worsnop, D. R., Collins, D. R., Tomlinson, J., and Baltensperger, U.: Relating hygroscopicity and composition of organic aerosol particulate matter, Atmos. Chem. Phys., 11, 1155–1165, doi:10.5194/acp-11-1155-2011, 2011.

Feiner, P. A. W. H. Brune, D. O. Miller, L. Zhang, R. C. Cohen, P. S. Romer, A. H. Goldstein, F. N. Keutsch, K. M. Skog, P. O. Wennberg, T. Nguyen, A. P. Teng, J. DeGouw, A. Koss, R. J. Wild, S. S. Brown, A. Guenther, E. Edgerton, K. Baumann, J. L. Fry, Testing atmospheric oxidation in an Alabama forest, submitted to J. Atmos. Sci.

Isaacman, G., N.M. Kreisberg, L.D. Yee, D.R. Worton, A.W.H. Chan, J.A. Moss, S.V. Hering, and A.H. Goldstein, Online derivatization for hourly measurements of gas- and particle-phase semi-volatile oxygenated organic compounds by thermal desorption aerosol gas chromatography (SV-TAG), Atmos. Meas. Tech., 7, 4417-4429, doi:10.5194/amt-7-4417-2014, 2014.

Jimenez, J. L., Canagaratna, M. R., Donahue, N. M., Prevot, A. S. H., Zhang, Q., Kroll, J. H., DeCarlo, P. F., Allan, J. D., Coe, H., Ng, N. L., Aiken, A. C., Docherty, K. S., Ulbrich, I. M., Grieshop, A. P., Robinson, A. L., Duplissy, J., Smith, J. D., Wilson, K. R., Lanz, V. A., Hueglin, C., Sun, Y. L., Tian, J., Laaksonen, A., Raatikainen, T., Rautiainen, J., Vaattovaara, P., Ehn, M., Kulmala, M., Tomlinson, J. M., Collins, D. R., Cubison, M. J., Dunlea, J., Huffman, J. A., Onasch, T. B., Alfarra, M. R., Williams, P. I., Bower, K., Kondo, Y., Schneider, J., Drewnick, F., Borrmann, S., Weimer, S., Demerjian, K., Salcedo, D., Cottrell, L., Griffin, R., Takami, A., Miyoshi, T., Hatakeyama, S., Shimono, A., Sun, J. Y., Zhang, Y. M., Dzepina, K., Kimmel, J. R., Sueper, D., Jayne, J. T., Herndon, S. C., Trimborn, A. M., Williams, L. R., Wood, E. C., Middlebrook, A. M., Kolb, C. E., Baltensperger, U., and Worsnop, D. R.: Evolution of Organic Aerosols in the Atmosphere, Science, 326, 1525–1529, doi:10.1126/science.1180353, 2009.

Lambe, A. T., Onasch, T. B., Massoli, P., Croasdale, D. R., Wright, J. P., Ahern, A. T., Williams, L. R., Worsnop, D. R., Brune, W. H., and Davidovits, P.: Laboratory studies of the chemical composition and cloud condensation nuclei (CCN) activity of secondary organic aerosol (SOA) and oxidized primary organic aerosol (OPOA), Atmos. Chem. Phys., 11, 8913–8928, doi:10.5194/acp-11-8913-2011, 2011.

Misztal, P. K., Guenther, A., and Goldstein, A. H.: Flux observations of isoprene oxidation products above forests point to potential role of leaf-surface reactions, in preparation, 2016.

Nguyen, T. B., Crounse, J. D., Teng, A. P., St. Clair, J. M., Paulot, F., Wolfe, G. M., and Wennberg, P. O.: Rapid deposition of oxidized biogenic compounds to a temperate forest, Proc. Natl. Acad. Sci. U. S. A., 112, E392–E401, doi:10.1073/pnas.1418702112, 2015.

Raatikainen, T., Vaattovaara, P., Tiitta, P., Miettinen, P., Rautiainen, J., Ehn, M., Kulmala, M., Laaksonen, A., and Worsnop, D. R.: Physicochemical properties and origin of organic groups detected in boreal forest using an aerosol mass spectrometer, Atmos. Chem. Phys., 10, 2063–2077, doi:10.5194/acp-10-2063-2010, 2010.

Sander R.: Compilation of Henry's Law Constants for Inorganic and Organic Species of Potential Importance in Environmental Chemistry (Max Planck Inst Chem, Mainz, Germany). http://www.henrys-law.org/henry-3.0.pdf, 1999.

Simon, H. and Bhave, P. V.: Simulating the degree of oxidation in atmospheric organic particles, Environ. Sci. Technol., 46, 331–9, 2012.

You, Y., Renbaum-Wolff, L., and Bertram, A. K.: Liquid-liquid phase separation in particles containing organics mixed with ammonium sulfate, ammonium bisulfate, ammonium nitrate or sodium chloride, Atmos. Chem. Phys., 13, 11 723–11 734, doi:10.5194/acp-13-11723-2013, 2013.

Wolfe, G. M., and Thornton, J. A.: The Chemistry of Atmosphere-Forest Exchange (CAFE) Model – Part 1: Model description and characterization. Atmos. Chem. Phys. 11, 1, 77-101, 2011.

Xu, L., Guo, H., Boyd, C. M., Klein, M., Bougiatioti, A., Cerully, K. M., Hite, J. R., Isaacman-VanWertz, G., Kreisberg, N. M., Knote, C., Olson, K., Koss, A., Goldstein, A. H., Hering, S. V., de Gouw, J., Baumann, K., Lee, S.-H., Nenes, A.,Weber, R. J., and Ng, N. L.: Effects of anthropogenic emissions on aerosol formation from isoprene and monoterpenes in the southeastern United States, Proc. Natl. Acad. Sci. U. S. A., 112, 37–42, doi:10.1073/pnas.1417609112, 2015.
