# Supplementary material for: On the implications of aerosol liquid water and phase separation for organic aerosol mass
Source: Atmos Chem Phys. Author manuscript; Available in PMC 2018 Aug 22. (PMC6104851; doi:10.5194/acp-17-343-2017)
Supplement: Supp [file NIHMS982683-supplement-Supp.zip › acp-17-343-2017-supplement-title-page.pdf]

Supplement of Atmos. Chem. Phys., 17, 343–369, 2017
http://www.atmos-chem-phys.net/17/343/2017/
doi:10.5194/acp-17-343-2017-supplement
© Author(s) 2017. CC Attribution 3.0 License.

Atmospheric Chemistry and Physics
Open Access

Supplement of

On the implications of aerosol liquid water and phase separation for organic aerosol mass

Havala O. T. Pye et al.

Correspondence to: Havala O. T. Pye (pye.havala@epa.gov)

- acp-17-343-2017-supplement-title-page.pdf
- acp-2016-719-supplement-version4.pdf
- acpdata
  - README.txt
  - figure11
    - S-A09_SOASsvdma_20130715_R0-Waterdata.OUT.0526hlawuporgwater.csv
    - S-A09_SOASsvdma_20130715_R0-Waterdata.OUT.0531base.csv
    - S-A09_SOASsvdma_20130715_R0-Waterdata.OUT.0531lle.csv
    - S-A09_SOASsvdma_20130715_R0-Waterdata.OUT.0616act.csv
    - S-A10-fRHwater_CTR_20130601_RA.OUT.0526hlawuporgwater.csv
    - S-A10-fRHwater_CTR_20130601_RA.OUT.0531base.csv
    - S-A10-fRHwater_CTR_20130601_RA.OUT.0531lle.csv
    - S-A10-fRHwater_CTR_20130601_RA.OUT.0616act.csv
    - S-A18-AMSMassElementalRatio_CTR_20130606_RB.OUT.0526hlawuporgwater.csv
    - S-A18-AMSMassElementalRatio_CTR_20130606_RB.OUT.0531base.csv
    - S-A18-AMSMassElementalRatio_CTR_20130606_RB.OUT.0531lle.csv
    - S-A18-AMSMassElementalRatio_CTR_20130606_RB.OUT.0616act.csv
  - figure13
    - S-A10-WSOC_CTR_20130601_RA.OUT.0531base.WSOC.csv
    - S-A10-WSOC_CTR_20130601_RA.OUT.0616act.WSOC.csv
  - figure14
    - WSOCg.OUT.0526hlawuporgwater.csv
    - WSOCg.OUT.0531base.csv
    - WSOCg.OUT.0531lle.csv
    - WSOCg.OUT.0616act.csv
  - figure6

    * ACSM_LRK_20130601_R0.OUT.0531base.csv
    * PMF_LRK_20130601_R0.OUT.0531base.csv
    * S-A15-HRAMS-ambientPMF_CTR_20130601_R0.OUT.0531base.csv
    * S-A15-HRAMS-ambient_CTR_20130601_R0.OUT.0531base.csv
  - figure789
    * CSN.OUT.0526hlawuporgwater.csv
    * CSN.OUT.0531base.csv
    * CSN.OUT.0531lle.csv
    * CSN.OUT.0616act.csv
    * IMPROVE.OUT.0526hlawuporgwater.csv
    * IMPROVE.OUT.0531base.csv
    * IMPROVE.OUT.0531lle.csv
    * IMPROVE.OUT.0616act.csv
    * SEARCH.OUT.0526hlawuporgwater.csv
    * SEARCH.OUT.0531base.csv
    * SEARCH.OUT.0531lle.csv
    * SEARCH.OUT.0616act.csv

The copyright of individual parts of the supplement might differ from the CC-BY 3.0 licence.
